# Duration of chemotherapy prior to chemoradiation affects survival outcomes for resected stage I‐II or unresected stage III pancreatic cancer

**DOI:** 10.1002/cam4.2326

**Published:** 2019-06-10

**Authors:** Sung J. Ma, Austin J. Iovoli, Gregory M. Hermann, Kavitha M. Prezzano, Anurag K. Singh

**Affiliations:** ^1^ Department of Radiation Medicine Roswell Park Comprehensive Cancer Center Buffalo NY; ^2^ Jacobs School of Medicine and Biomedical Sciences University at Buffalo, The State University of New York Buffalo NY

**Keywords:** induction, locally advanced, pancreas, radiation, resectable

## Abstract

**Background:**

For resected early stage pancreatic cancer, RTOG 9704 evaluated the outcome of 3 weeks of postoperative chemotherapy (C) followed by chemoradiation (CRT) and further C. For unresectable locally advanced pancreatic cancer, a recent literature review of prospective studies showed that the duration of induction C prior to CRT can impact survival. However, the ideal duration of C prior to CRT remains unclear for these patient cohorts. This National Cancer Database (NCDB) study was performed to compare the outcome of various durations of C prior to CRT.

**Methods:**

The NCDB was queried for resected primary stage I‐II, cT1‐3N0‐1M0, and unresected stage III, cT4N0‐1M0 pancreatic adenocarcinoma treated with C + CRT (2004‐2015). Cohorts I‐II and III included stage I‐II and stage III cases, respectively. Patients were stratified by short (short C) and long duration (long C) of chemotherapy based on their median durations. Baseline patient, tumor, and treatment characteristics were examined. The primary endpoint was overall survival (OS). Kaplan‐Meier analysis, multivariable Cox proportional hazards method, and propensity score matching were used.

**Results:**

Among 1577 patients, cohort I‐II had 839 patients and cohort III had 738 patients. The longer duration of chemotherapy prior to CRT showed improved OS in the multivariate analysis in both cohort I‐II (hazards ratio [HR] 0.72, *P* < 0.001) and cohort III (HR 0.83, *P* = 0.03). Using 1:1 propensity score matching, 610 patients for cohort I‐II and 542 patients for cohort III were matched. After matching, long C remained statistically significant for improved OS compared with short C in both cohort I‐II (median OS 26.1 vs 21.9 months; *P* = 0.003) and cohort III (median OS 16.7 vs 14.2; *P* = 0.02).

**Conclusion:**

Our NCDB study using propensity score‐matched analysis showed a survival benefit for using the longer duration of chemotherapy compared to the shorter duration for both resected stage I‐II and unresected stage III pancreatic cancer.

## 1 INTRODUCTION

Pancreatic cancer is an aggressive neoplasm with a median survival of approximately 1 year.^1^ For early stage pancreatic cancer, definitive management is attained through surgical resection followed by adjuvant chemotherapy (C) with or without chemoradiation (CRT). Several key studies have drawn conflicting conclusions regarding the use of CRT in this population.^2^, ^3^, ^4^ RTOG 9704 evaluated the outcome in resected pancreatic cancer of 3 weeks of postoperative C followed by CRT and further C. This trial demonstrated a potential survival advantage of patients receiving adjuvant C and CRT, but was limited in study design by patients receiving a short duration of C prior to CRT and by prolonged interruptions to C during treatment.^3^

Optimal management for unresectable locally advanced pancreatic cancer (LAPC) using C and CRT is also under investigation. Several studies have shown promising efficacy in LAPC treatment utilizing regimens involving induction C followed by CRT.^5^, ^6^, ^7^ A recent literature review of LAPC prospective studies showed a survival benefit of induction C and CRT together over C alone when the induction C lasted at least 3 months.^8^ Similarly, another study by Faisal et al found a trend toward improved survival in patients with LAPC who received more than two cycles of C prior to CRT.^9^

For both resected early stage pancreatic cancer and unresectable LAPC, the ideal duration of chemotherapy prior to CRT remains unclear. This National Cancer Database (NCDB) study was performed using aggregated hospital registry data to compare outcomes between shorter and longer durations of chemotherapy prior to CRT for pancreatic cancer.

## 2 METHODS

### 2.1 Patient population

The NCDB registry was queried for patients with pancreatic adenocarcinoma diagnosed between 2004 and 2015 (the most recent dataset available at the time of this study). The NCDB is a national cancer database capturing approximately 70% of the cancer incidence cases in the United States. It also includes over 30 million historical records.^10^ This dataset is de‐identified and was exempt from institutional review board's review.

**Figure 1:**
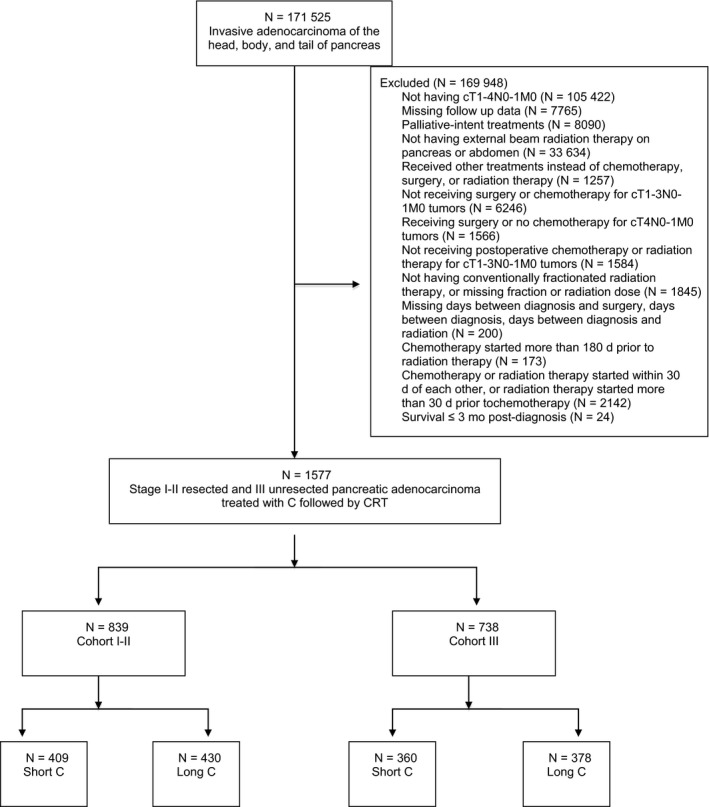
Flow diagram for patient selection. C: chemotherapy; CRT: chemoradiation

Detailed criteria for patient selection are shown in Figure 1. From our initial query, we selected two patient cohorts: cohorts I‐II for resected stage I‐II, clinical T1‐3N0‐1M0 and cohort III for unresected stage III, clinical T4N0‐1M0 pancreatic adenocarcinoma. Cohort I‐II included patients who had been treated with curative‐intent resection and adjuvant C followed by adjuvant CRT. Cohort III included those who had been treated with curative‐intent induction C followed by CRT. Stage I‐III diseases in 2004‐2015 were based on American Joint Committee on Cancer 6th and 7th editions definitions.

All patients received conventionally fractionated radiation therapy (CFRT). To address variability in dose fractionation among hospitals for resected stage I‐II and unresected stage III pancreatic cancer, CFRT was defined as 45‐60 Gy using 1.8‐2.0 Gy/fraction for cohort I‐II and 45‐70 Gy using 1.8‐2.5 Gy/fraction for cohort III.^11^, ^12^ For cohort I‐II, Whipple surgery was defined as local or partial pancreatectomy and duodenectomy with partial gastrectomy. Whipple‐variant surgery was characterized as partial pancreatectomy with duodenectomy, total pancreatectomy alone, or total pancreatectomy with subtotal gastrectomy or duodenectomy.^13^ For both cohorts, those treated with C or radiation therapy within 30 days of each other were considered to have received concurrent CRT alone and were excluded from our analysis. Patients treated with C within 31‐180 days prior to the radiation therapy were considered to have received postoperative C followed by CRT for cohort I‐II and induction C followed by CRT for cohort III.^14^ Patients treated with C more than 180 days prior to radiation therapy were excluded.

Exclusion criteria were incomplete follow‐up or vital status data, metastatic pancreatic cancer, palliative‐intent treatments, neoadjuvant C or radiation, missing radiation dose or fractionation information, having no C or radiation therapy, having surgery for cohort III, incomplete data on the number of days between diagnosis and treatments, and patients with post‐diagnosis survival duration of less than 3 months.

For cohort I‐II, baseline characteristics for analysis were treatment facility type, age, gender, race, insurance, household income, residential setting, Charlson‐Deyo comorbidity score (CDS), year of diagnosis, primary tumor site within pancreas, tumor grade, tumor size, clinical T and N stages, pathologic T and N stages, surgery type, surgical margin, single‐ vs multi‐agent C, total radiation dose and fractionation, and the number of days between the onset of C and CRT. For cohort III, its baseline characteristics were similar to the aforementioned variables, except for pathologic T and N stages, surgery type, surgical margin, since no surgery was performed in this cohort. For cohort I‐II, surgical margin was categorized as either negative (R0) or positive (R1, R2, positive margin not otherwise specified). Patients were stratified by age ≥65 years or <65 years for both cohorts, tumor size <3.0 cm or ≥3.0 cm for cohort I‐II, and tumor size <3.8 cm or ≥3.8 cm for cohort III based on their median values. For each cohort, short (short C) and long (long C) duration of chemotherapy prior to CRT were determined based on the median values of the number of days between the start of C and radiation therapy. The household income level of each patient's residential area was according to the 2012 American Community Survey data adjusted for inflation (the most recent data at the time of this study), and it was categorized by above or below the median value of $48 000.

Pertinent prognostic factors, such as type and duration of chemotherapy, and patient performance status are unavailable in the NCDB. Other outcomes, such as local and distant failure and toxicity data are also unavailable in the dataset. For cohort I‐II, CA 19‐9 factor was excluded for analysis, since 439 patients (52.3%) had missing values and 176 patients (21.0%) had unknown values above 98 U/mL. For cohort III, tumor grade was excluded for analysis, since 583 patients (79.0%) had missing values. For this cohort, 288 patients (39.0%) had missing values for CA 19‐9 factor and another 264 patients (35.8%) had unknown values above 98 U/mL. CA 19‐9 level was also excluded for analysis in cohort III. The primary endpoint was overall survival (OS), defined as time between the diagnosis and the last follow‐up or death.

### 2.2 Statistical analysis

Overall survival was examined using Kaplan‐Meier and log‐rank tests. Categorical and continuous variables between the short and long C groups were compared using Fisher's exact and Mann‐Whitney U tests, respectively. Logistic regression univariate (UVA) and multivariate analyses (MVA) were used to determine predictors for the receipt of long C and were indicated as odds ratio (OR). Cox proportional hazard UVA and MVA were used to determine predictors for the OS and were indicated as hazards ratio (HR). MVA models were constructed using all statistically significant factors from UVA and were finalized based on a backward stepwise elimination. Treatment interactions with other variables were examined using Cox MVA by adding interaction terms.^15^

In order to reduce selection bias, propensity score matching was performed based on baseline characteristics. For cohort I‐II, these characteristics include facility type, age, CDS, tumor grade, tumor size, year of diagnosis, pathologic T and N stages, surgery type, surgical margin, single‐ vs multi‐agent chemotherapy use, and total radiation dose. For cohort III, baseline characteristics for matching included aforementioned variables from cohort I‐II in addition to clinical N stage, except for tumor grade, pathologic T and N stages, surgery type, and surgical margin. Additional variables were considered for matching if they were statistically significant in Cox MVA for OS. All matching was performed in a 1:1 ratio without replacements based on the nearest neighbor method with a caliper distance of 0.2 of the standard deviation of the logit of the propensity score.^16^ MatchIt package (version 3.0.1) was used for matching. After matching, matched‐sample Cox UVA was performed to evaluate the survival benefit of long C. All aforementioned analyses were performed using R software (version 3.4.3, R Foundation for Statistical Computing, Vienna, Austria). All p values were two‐sided and those less than 0.05 were considered statistically significant.

## 3 RESULTS

### 3.1 Cohort I‐II

A total of 1577 patients with clinical stage I‐III pancreatic adenocarcinoma met the inclusion criteria and were identified. Of those, 839 patients with resected stage I‐II, clinical T1‐3N0‐1M0 pancreatic adenocarcinoma received postoperative C followed by CRT. The median value of the number of days between the onset of C and CRT in this cohort was 70 days. Short C and long C were stratified by <70 (n = 409) and ≥70 days (n = 430), respectively. Short C had a median of 45 days (interquartile range [IQR] 35‐57) and long C had a median of 109 days (IQR 85‐138; *P* < 0.001) between the start of C and CRT. The majority of patients had stage II pathologic T3N1M0 moderately or poorly differentiated adenocarcinoma of the pancreatic head (Table 1). The long C group had more patients with negative surgical margins and who were treated with single‐agent chemotherapy. Other variables were well balanced.

**Table 1:** Baseline characteristics for cohort I‐II, before and after matching

	Before matching					After matching				
	Short C		Long C		*P*	Short C		Long C		*P*
	N	%	N	%		N	%	N	%	
Facility					0.94					0.87
Nonacademic	259	63	272	63		195	64	198	65	
Academic	145	35	154	36		110	36	107	35	
NA	5	1	4	1		0	0	0	0	
Age					0.19					0.57
<65	205	50	236	55		155	51	163	53	
≥65	204	50	194	45		150	49	142	47	
NA	0	0	0	0		0	0	0	0	
Gender					0.58					
Female	197	48	216	50						
Male	212	52	214	50						
NA	0	0	0	0						
Race					0.06					
White	362	89	362	84						
Black	35	9	41	10						
Other	11	3	25	6						
NA	1	0	2	0						
Insurance					0.90					
None	11	3	13	3						
Nonprivate	211	52	215	50						
Private	184	45	198	46						
NA	3	1	4	1						
Income					0.88					
Above median	271	66	290	67						
Below median	132	32	137	32						
NA	6	1	3	1						
Residential setting					0.30					
Metro	326	80	358	83						
Urban	62	15	50	12						
Rural	7	2	9	2						
NA	14	3	13	3						
Charlson‐Deyo Score					0.88					1
0‐1	384	94	405	94		284	93	285	93	
≥2	25	6	25	6		21	7	20	7	
NA	0	0	0	0		0	0	0	0	
Year of diagnosis					0.67					0.84
2004‐2007	21	5	23	5		14	5	12	4	
2008‐2011	216	53	214	50		152	50	148	49	
2012‐2015	172	42	193	45		139	46	145	48	
NA	0	0	0	0		0	0	0	0	
Primary tumor site					0.36					
Head	331	81	364	85						
Body	35	9	30	7						
Tail	43	11	36	8						
NA	0	0	0	0						
Tumor grade					0.28					0.24
Well diff	33	8	39	9		26	9	31	10	
Mod diff	207	51	216	50		174	57	162	53	
Poor diff	129	32	139	32		95	31	108	35	
Other	11	3	4	1		10	3	4	1	
NA	29	7	32	7		0	0	0	0	
Tumor size					0.40					0.87
<3.0	173	42	168	39		125	41	122	40	
≥3.0	229	56	251	58		180	59	183	60	
NA	7	2	11	3		0	0	0	0	
Clinical T stage					0.06					
1	56	14	72	17						
2	150	37	179	42						
3	203	50	179	42						
NA	0	0	0	0						
Clinical N stage					0.06					
0	260	64	301	70						
1	149	36	129	30						
NA	0	0	0	0						
Pathologic T stage					0.36					0.06
0	0	0	1	0		0	0	1	0	
1	18	4	16	4		15	5	11	4	
2	36	9	50	12		23	8	34	11	
3	331	81	344	80		260	85	258	85	
4	9	2	5	1		7	2	1	0	
NA	15	4	14	3		0	0	0	0	
Pathologic N stage					0.45					0.43
0	83	20	98	23		62	20	71	23	
1	304	74	314	73		243	80	234	77	
NA	22	5	18	4		0	0	0	0	
Surgery					0.76					0.72
Whipple‐variant	115	28	127	30		90	30	96	31	
Whipple	205	50	218	51		154	50	155	51	
Other	89	22	85	20		61	20	54	18	
NA	0	0	0	0		0	0	0	0	
Surgical margin					0.005					0.62
Negative	287	70	335	78		236	77	242	79	
Positive	118	29	87	20		69	23	63	21	
NA	4	1	8	2		0	0	0	0	
Chemotherapy					0.03					0.46
Single‐agent	218	53	261	61		166	54	176	58	
Multi‐agent	191	47	169	39		139	46	129	42	
NA	0	0	0	0		0	0	0	0	
Total radiation dose (Gy)					0.16					0.73
Median	50.4		50.4			50.4		50.4		
IQR	50.4‐50.4		50.4‐50.4			50.4‐50.4		50.4‐50.4		
Fraction					0.17					
Median	28		28							
IQR	28‐28		28‐28							

Abbreviation: IQR, interquartile range.

On logistic regression MVA, patients who were neither Caucasian nor African‐American (OR 2.36, *P* = 0.02), and who had received a total radiation dose >54 Gy (OR 3.25, *P* = 0.002) were more likely to receive long C. In addition, patients with positive surgical margins (OR 0.57, *P* < 0.001) were less likely to receive long C. No other variables were statistically significant for the receipt of long C.

On Cox MVA (Table 2), treatments at academic facilities (HR 0.73, *P* = 0.001) and long C (HR 0.72, *P* < 0.001) showed improved OS. Having moderately (HR 1.60, *P* = 0.01) or poorly (HR 2.21, *P* < 0.001) differentiated tumors, larger tumors (HR 1.48, *P* < 0.001), positive pathologic nodal status (HR 1.37, *P* = 0.009), and positive surgical margins (HR 1.33, *P* = 0.005) were associated with worse mortality. After Cox MVA, there was no treatment interaction with age (*P* = 0.60), CDS (*P* = 0.42), or year of diagnosis (2008‐2011, *P* = 0.46; 2012‐2015, *P* = 0.91).

**Table 2:** Cox UVA and MVA for cohort I‐II

Variable	Cox UVA			Cox MVA		
	HR	95% CI	*P*	HR	95% CI	*P*
Facility						
Nonacademic	1	Ref		1	Ref	
Academic	0.74	0.63‐0.88	<0.001	0.73	0.61‐0.88	0.001
Age						
<65	1	Ref				
≥65	1.08	0.92‐1.27	0.36			
Gender						
Female	1	Ref				
Male	1.06	0.90‐1.25	0.48			
Race						
White	1	Ref				
Black	0.95	0.71‐1.27	0.73			
Other	0.93	0.61‐1.41	0.73			
Insurance						
None	1	Ref				
Nonprivate	0.85	0.53‐1.34	0.48			
Private	0.71	0.44‐1.13	0.15			
Income						
Above median	1	Ref		1	Ref	
Below median	1.20	1.01‐1.42	0.04	1.16	0.96‐1.41	0.12
Residential setting						
Metro	1	Ref		1	Ref	
Urban	0.93	0.73‐1.18	0.54			
Rural	1.88	1.10‐3.20	0.02	1.42	0.79‐2.56	0.24
Charlson‐Deyo Score						
0‐1	1	Ref				
≥2	1.10	0.78‐1.54	0.60			
Year of diagnosis						
2004‐2007	1	Ref		1	Ref	
2008‐2011	0.77	0.56‐1.06	0.11			
2012‐2015	0.68	0.48‐0.95	0.03	0.93	0.61‐1.43	0.75
Primary tumor site						
Head	1	Ref				
Body	0.97	0.72‐1.30	0.83			
Tail	1.10	0.84‐1.44	0.50			
Tumor grade						
Well diff	1	Ref		1	Ref	
Mod diff	1.50	1.07‐2.10	0.02	1.6	1.11‐2.31	0.01
Poor diff	1.92	1.36‐2.71	<0.001	2.21	1.52‐3.23	<0.001
Other	1.64	0.79‐3.39	0.18			
Tumor size						
<3.0	1	Ref		1	Ref	
≥3.0	1.65	1.39‐1.96	<0.001	1.48	1.23‐1.78	<0.001
Pathologic T stage						
0	1	Ref				
1	0.25	0.034‐1.88	0.18			
2	0.29	0.040‐2.11	0.22			
3	0.39	0.054‐2.76	0.34			
4	0.39	0.049‐3.13	0.38			
Pathologic N stage						
0	1	Ref		1	Ref	
1	1.49	1.20‐1.84	<0.001	1.37	1.08‐1.73	0.009
Surgery						
Whipple‐variant	1	Ref				
Whipple	0.87	0.72‐1.05	0.14			
Other	0.96	0.77‐1.21	0.74			
Surgical margin						
Negative	1	Ref		1	Ref	
Positive	1.37	1.14‐1.65	<0.001	1.33	1.09‐1.63	0.005
Chemotherapy						
Single‐agent	1	Ref				
Multi‐agent	1.02	0.86‐1.20	0.86			
Total radiation dose (Gy)						
Per 1 Gy increase	0.9997	0.97‐1.03	0.98			
Chemo duration						
Short C	1	Ref		1	Ref	
Long C	0.79	0.67‐0.92	0.004	0.72	0.60‐0.86	<0.001

Abbreviation: HR, hazards ratio.

The overall median follow‐up in cohort I‐II was 39.5 months (IQR 26.3‐58.2). The short C group had a median follow‐up of 37.8 months (IQR 26.5‐58.7) and the long C group had that of 40.3 months (IQR 26.3‐58.1). The median OS for cohort I‐II was 21.9 months (IQR 15.2‐36.6) for the short C group and 25.9 months (IQR 17.6‐41.9) for the long C group (log‐rank *P* = 0.003). OS at 2 years was 49.4% for the short C group and 59.5% for the long C group.

**Figure 2:**
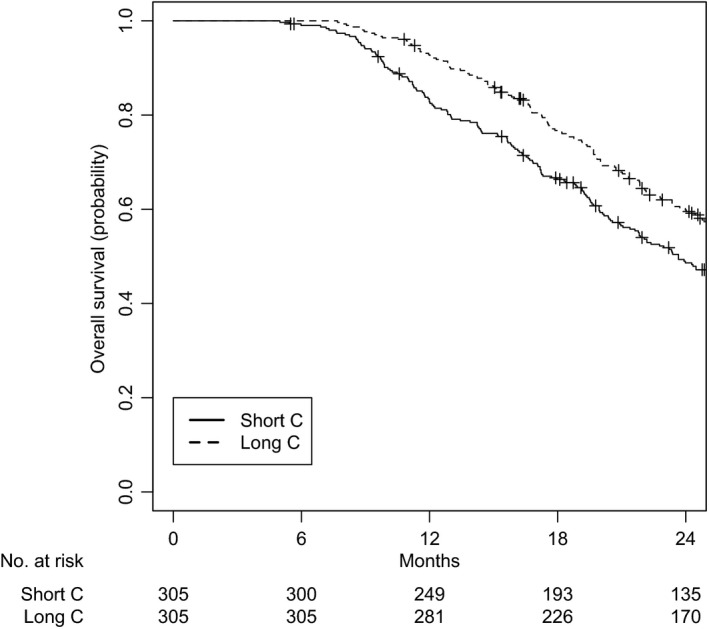
Overall survival for cohort I‐II, after matching. C: chemotherapy

A total of 610 patients were matched, with 305 patients in each group. All variables were well balanced (Table 1). The median follow‐up was 36.6 months (IQR 24.8‐52.9) for the short C group and 41.0 months (IQR 27.5‐56.0) for the long C group. The median OS was 21.9 months (IQR 14.5‐36.5) for the short C group and 26.1 months (IQR 17.7‐42.1) for the long C group (log‐rank *P* = 0.003). OS at 2 years was 48.6% for the short C group and 60.0% for the long C group (Figure 2).

### 3.2 Cohort III

A total of 738 patients had unresected stage III, clinical T4N0‐1M0 pancreatic adenocarcinoma and were included in cohort III. The median number of days between the onset of C and CRT in this cohort was 90 days. Short C and long C were stratified by <90 (n = 360) and ≥90 days (n = 378), respectively. Short C had a median of 67 days (IQR 50‐77) and long C had a median of 119 days (IQR 103‐140; *P* < 0.001) between the start of C and CRT. The majority of patients had clinical T4N0 adenocarcinoma of the pancreatic head (Table 3). The long C group was more likely to include patients treated at academic facilities, diagnosed between 2012 and 2015, and who had received multi‐agent chemotherapy.

**Table 3:** Baseline characteristics for cohort III, before and after matching

	Before matching					After matching				
	Short C		Long C		*P*	Short C		Long C		*P*
	N	%	N	%		N	%	N	%	
Facility					0.003					0.67
Nonacademic	218	61	188	50		153	56	147	54	
Academic	138	38	186	49		118	44	124	46	
NA	4	1	4	1		0	0	0	0	
Age					0.71					0.61
<65	184	51	188	50		137	51	130	48	
≥65	176	49	190	50		134	49	141	52	
NA	0	0	0	0		0	0	0	0	
Gender					0.06					
Female	171	48	207	55						
Male	189	53	171	45						
NA	0	0	0	0						
Race					0.42					
White	291	81	317	84						
Black	54	15	44	12						
Other	11	3	11	3						
NA	4	1	6	2						
Insurance					0.73					
None	8	2	6	2						
Nonprivate	190	53	193	51						
Private	160	44	175	46						
NA	2	1	4	1						
Income					0.59					
Above median	230	64	249	66						
Below median	128	36	126	33						
NA	2	1	3	1						
Residential setting					0.71					
Metro	288	80	314	83						
Urban	49	14	44	12						
Rural	7	2	8	2						
NA	16	4	12	3						
Charlson‐Deyo Score					0.11					1
0‐1	349	97	357	94		263	97	262	97	
≥2	11	3	21	6		8	3	9	3	
NA	0	0	0	0		0	0	0	0	
Year of diagnosis					<0.001					0.92
2004‐2007	38	11	21	6		15	6	16	6	
2008‐2011	182	51	137	36		130	48	125	46	
2012‐2015	140	39	220	58		126	46	130	48	
NA	0	0	0	0		0	0	0	0	
Primary tumor site					0.47					
Head	251	70	254	67						
Body	98	27	116	31						
Tail	11	3	8	2						
NA	0	0	0	0						
Tumor size					0.32					0.44
<3.8	151	42	179	47		127	47	137	51	
≥3.8	173	48	174	46		144	53	134	49	
NA	36	10	25	7		0	0	0	0	
Clinical N stage					0.10					0.37
0	211	59	245	65		171	63	182	67	
1	149	41	133	35		100	37	89	33	
NA	0	0	0	0		0	0	0	0	
Chemotherapy					<0.001					0.62
Single‐agent	119	33	77	20		71	26	65	24	
Multi‐agent	241	67	301	80		200	74	206	76	
NA	0	0	0	0		0	0	0	0	
Total radiation dose (Gy)					0.66					0.08
Median	50.4		50.4			50.4		50.4		
IQR	50.4‐54		50.4‐54			50.4‐54.0		50.4‐54.3		
Fraction					0.16					
Median	28		28							
IQR	27‐30		27‐29							

Abbreviation: IQR, interquartile range.

On logistic MVA, patients treated at academic facilities (OR 1.48, *P* = 0.01), diagnosed between 2012‐2015 (OR 2.65, *P* < 0.001), and who were treated with multi‐agent chemotherapy (OR 1.66, *P* = 0.004) were associated with the receipt of long C.

On Cox MVA (Table 4), treatments at academic facilities (HR 0.81, *P* = 0.009), being diagnosed between 2012 and 2015 (HR 0.73, *P* = 0.04), the use of multi‐agent chemotherapy (HR 0.77, *P* = 0.005), and long C duration (HR 0.83, *P* = 0.03) were associated with improved survival. After Cox MVA, no treatment interaction was observed with age (*P* = 0.39), CDS (0.65), year of diagnosis (2008‐2011, *P* = 0.91; 2012‐2015, *P* = 0.77).

**Table 4:** Cox UVA and MVA for cohort III

Variable	Cox UVA			Cox MVA		
	HR	95% CI	P	HR	95% CI	*P*
Facility						
Nonacademic	1	Ref		1	Ref	
Academic	0.76	0.65‐0.89	<0.001	0.81	0.69‐0.95	0.009
Age						
<65	1	Ref				
≥65	1.04	0.89‐1.21	0.64			
Gender						
Female	1	Ref		1	Ref	
Male	1.19	1.02‐1.39	0.03	1.10	0.93‐1.30	0.25
Race						
White	1	Ref				
Black	0.86	0.68‐1.10	0.23			
Other	0.86	0.54‐1.36	0.52			
Insurance						
None	1	Ref				
Nonprivate	1.07	0.57‐2.02	0.82			
Private	1.07	0.57‐2.01	0.83			
Income						
Above median	1	Ref				
Below median	1.05	0.90‐1.24	0.52			
Residential setting						
Metro	1	Ref				
Urban	0.996	0.79‐1.26	0.98			
Rural	1.35	0.80‐2.25	0.26			
Charlson‐Deyo Score						
0‐1	1	Ref				
≥2	1.08	0.75‐1.57	0.67			
					
2004‐2007	1	Ref		1	Ref	
2008‐2011	0.95	0.71‐1.28	0.75			
2012‐2015	0.67	0.50‐0.89	0.007	0.73	0.54‐0.98	0.04
Primary tumor site						
Head	1	Ref				
Body	0.87	0.73‐1.04	0.12			
Tail	1.25	0.78‐2.01	0.35			
Tumor size (cm)						
<3.8	1	Ref		1	Ref	
≥3.8	1.20	1.02‐1.41	0.03	1.17	0.99‐1.38	0.06
Clinical N stage						
0	1	Ref				
1	1.15	0.98‐1.34	0.09			
Chemotherapy						
Single‐agent	1	Ref		1	Ref	
Multi‐agent	0.69	0.58‐0.82	<0.001	0.77	0.65‐0.92	0.005
Total radiation dose (Gy)						
Per 1 Gy increase	0.99	0.97‐1.00	0.12			
Chemo duration						
Short C	1	Ref		1	Ref	
Long C	0.75	0.64‐0.88	<0.001	0.83	0.71‐0.98	0.03

Abbreviation: HR, hazards ratio.

The overall median follow‐up was 24.3 months (IQR 16.2‐38.0) for cohort III. The median follow‐up was 23.8 months (IQR 12.9‐33.0) for the short C group and 24.6 months (IQR 17.7‐40.4) for the long C group. The median OS was 14.0 months (IQR 9.5‐22.0) for the short C group and 17.4 months (13.0‐24.3) for the long C group (log‐rank *P* < 0.001). OS at 2 years was 23.6% for the short C group and 30.2% for the long C group.

**Figure 3:**
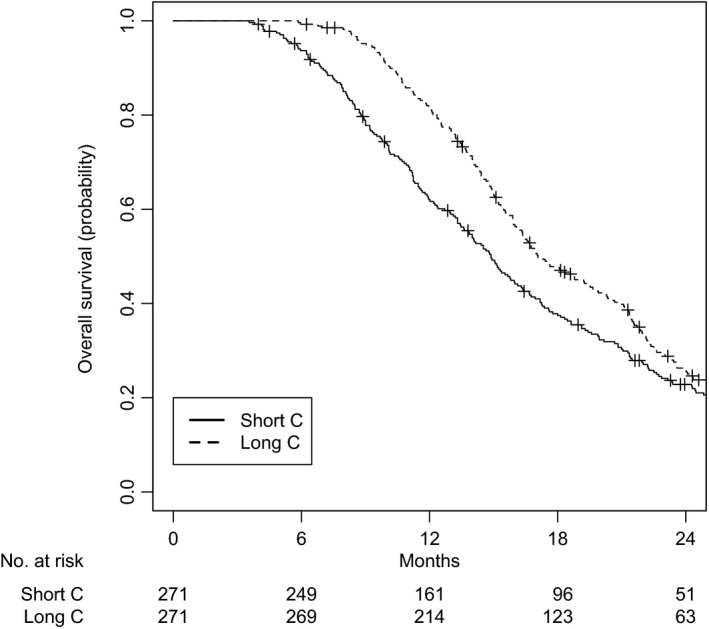
Overall survival for cohort III, after matching**.** C: chemotherapy

A total of 542 patients were matched, with 271 patients in each group. All variables were well balanced (Table 3). The median follow‐up was 23.5 months (IQR 11.3‐32.3) for the short C group and 22.5 months (IQR 13.9‐42.7) for the long C group. The median OS was 14.2 months (IQR 9.2‐21.7) for the short C group and 16.7 months (IQR 13.0‐23.3) for the long C group (log‐rank *P* = 0.02). OS at 2 years was 22.8% for the short C group and 26.3% for the long C group (Figure 3).

## 4 DISCUSSION

To the best of our knowledge, this study is the first to show that longer duration of chemotherapy prior to CRT improves survival outcomes for stage I‐II and stage III pancreatic cancer using aggregated hospital registry data. We found that longer duration of chemotherapy showed improved OS in the multivariable analysis in both cohort I‐II (HR 0.72, *P* < 0.001) and cohort III (HR 0.83, *P* = 0.03). After 1:1 propensity score matching, long C remained statistically significant for improved OS compared with short C in both cohort I‐II (median OS 26.1 vs 21.9 months; 2‐year OS 60.0% vs 48.6%; *P* = 0.003) and cohort III (median OS 16.7 vs 14.2 months; 2‐year OS 26.3% vs 22.8%; *P* = 0.02).

Reasons for survival benefit in longer duration of C are not clear. It could be the case that patients who are able to receive longer courses of C are better able to tolerate the associated toxicities. As performance status is unable to be assessed in the NCDB, it is likely that patients receiving long C had more robust performance compared to those unable to tolerate long C. Alternatively, those who received a shorter duration of chemotherapy could have experienced more treatment‐related adverse events. However, since they all received radiation therapy, such adverse events may have been reversible prior to the initiation of radiation. A longer course of chemotherapy may have treated micrometastasis more effectively prior to the local treatment using radiation which is supported by our finding that two‐thirds of stage I‐II patients with cN0 disease were upstaged to pN1. It is also possible that those with shorter chemotherapy duration have less responsive tumor biology. Among patients with stage I‐II pancreatic cancer, scans for restaging are not routinely performed post‐chemotherapy and pre‐CRT to determine the duration of chemotherapy. The duration of chemotherapy was likely determined prior to its initiation. Among patients with stage III pancreatic cancer, scans for restaging post‐chemotherapy and pre‐CRT are sometimes performed, but likely not as routinely as post‐CRT. However, such data are not captured in the NCDB.

In RTOG 9704, patients with stage I‐II pancreatic cancer were treated with 3 weeks of C, followed by a 1‐2 week treatment break, before starting CRT.^3^ Similarly in the EORTC‐FFCD‐GERCOR trial, patients received two cycles (for a total of 8 weeks) of C prior to CRT.^2^ Neither of these trials demonstrated a survival benefit in favor of their experimental arm. Our study demonstrated that patients with resected, stage I‐II pancreatic cancer who received more than 70 days of C prior to CRT had better survival, suggesting patients included in RTOG 9704 and the EORTC‐FFCD‐GERCOR trials may have not received a long enough course of C before initiating CRT to demonstrate improved survival.

It has been previously demonstrated that FOLFIRINOX is superior to gemcitabine for metastatic disease and was recently shown to significantly increase survival in the adjuvant setting.^17^, ^18^ Despite this, in the adjuvant setting, the FOLFIRINOX group had fewer patients receive all planned cycles of chemotherapy and a greater number of patients experience a delay and even a chemotherapy dose modification. Therefore, it is possible that our findings result from improved survival from the more active, but increasingly toxic FOLFIRINOX regimen. It is important to note that after matching, both groups had equal proportion of patients receiving multi‐agent chemotherapy. Our findings are further supported by MVA showing improved survival with multi‐agent chemotherapy and treatment occurring after publication of Conroy et al in 2011.

Improved OS with longer duration of C was found in unresected LAPC as well. This is consistent with a single‐institution retrospective report and a large meta‐analysis examining outcomes of induction C followed by CRT.^8^, ^9^ In the Johns Hopkins experience, treating patients with at least three cycles of C prior to CRT trended toward better OS.^9^ Similarly, the meta‐analysis noted a significant OS benefit when induction C lasted at least 3 months, which is the same threshold for improved survival found in our study.^8^

Due to the nature of a national registry‐based study, these results are limited by incomplete patient information and errors in documentation. One area particularly relevant to our report is the lack of toxicity outcomes in the NCDB. It is possible we found worse survival associated with shorter C because some patients experienced toxicities that required early discontinuation of C prior to CRT. However, without toxicity information this is difficult to assess. Further, the NCDB does not contain data on response to treatment, therefore it is unknown if patients with a poor response to chemotherapy also required early discontinuation of C prior to CRT. Despite the propensity score matching, we agree with multilevel selection bias as inherent limitations of NCDB and unavailable variables, such as performance status, types, and number of completed cycles of chemotherapy. Importantly, patient populations in rural, smaller hospitals are less likely captured by NCDB, and NCDB reports may not be representative of their clinical outcomes. In spite of these drawbacks, the NCDB contains the majority of cancer patients treated in the United States and provides a large cohort not otherwise accessible through single‐institution experiences. Ongoing clinical trials looking at various C and CRT combinations for treating pancreatic cancer, such as RTOG 0848, should provide greater insight into the optimal management for this challenging population.

We believe this is the first study using the NCDB to evaluate the ideal duration of chemotherapy prior to CRT for stage I‐II and stage III pancreatic cancer. Our study using propensity score‐matched analysis showed a significant survival benefit for patients who received a longer duration of chemotherapy compared to those undergoing a shorter duration course. This survival benefit was demonstrated in both resected stage I‐II and unresected stage III pancreatic cancer patients. Further prospective studies investigating the optimal length of chemotherapy in the management of pancreatic cancer are warranted.

## CONFLICT OF INTEREST

All authors declare that they have no competing interests.

## Data Availability

The data we used in this study, NCDB, are third party data from the American College of Surgeons. The data are available to researchers from the American Cancer Society or any Commission‐on‐Cancer accredited cancer programs. Data access request can be made following the instructions on https://www.facs.org/quality-programs/cancer/ncdb. The authors confirm they did not have any special access privileges to these data.
